# Supplementary material for: A pumpless liver-adipose model for studying metabolic dysfunction and drug responses
Source: PLoS One. 2026 May 8;21(5):e0345524. doi: 10.1371/journal.pone.0345524 (PMC13155575; doi:10.1371/journal.pone.0345524)
Supplement: S1 File — (DOCX) [file pone.0345524.s001.docx]

**Supporting information file**

**A pumpless liver-adipose model for studying metabolic dysfunction and drug responses**

**Zeinab Ebrahimian^1,2^ , Fatemeh Kalalinia^3,4^ , Amir Reza Ameri^2,5^ , Hossein Hosseinzadeh^1,6*^ , Bibi Marjan Razavi^1,7^ , and Seyed Ali Mousavi Shaegh^2,8,9,10*^**

^1^Department of Pharmacodynamics and Toxicology, School of Pharmacy, Mashhad University of Medical Sciences, Mashhad, Iran

^2^Laboratory for Microfluidics and Medical Microsystems, Research Institute for Medical Sciences, Mashhad University of Medical Sciences, Mashhad, Iran

^3^Biotechnology Research Center, Pharmaceutical Technology Institute, Mashhad University of Medical Sciences, Mashhad, Iran

^4^Department of Pharmaceutical Biotechnology, School of Pharmacy, Mashhad University of Medical Sciences, Mashhad, Iran

^5^School of Biomedical Engineering, University of British Columbia, Vancouver, Canada

^6^Pharmaceutical Research Center, Department of Pharmacodynamics and Toxicology, School of Pharmacy, Mashhad University of Medical Sciences, Mashhad, Iran

^7^Targeted Drug Delivery Research Center, Department of Pharmacodynamics and Toxicology, School of Pharmacy, Mashhad University of Medical Sciences, Mashhad, Iran

^8^Department of Biomedical Engineering, Mashhad University of Medical Sciences, Mashhad, Iran

^9^Clinical Research Unit, Ghaem Hospital, Mashhad University of Medical Sciences, Mashhad, Iran

^10^Orthopedic Research Center, Mashhad University of Medical Sciences, Mashhad, Iran

***Correspondence:**Hossein Hosseinzadeh, Seyed Ali Mousavi Shaegh
[hosseinzadehh@mums.ac.ir](mailto:hosseinzadehh@mums.ac.ir) (HH), Mousavisha@mums.ac.ir (SAMS)

**Keywords: Orgon-on-chip, Olanzapine, Chlorogenic acid, Metformin, Adipocyte–hepatocyte interaction.**

**S1 Table: Raw data for metabolic analyses.**

A- Oil Red O assay:

1- Quantitative Oil Red O assay of intracellular lipid content of different treatment conditions on 3T3-L1 in the perfusion-based co-culture (PBCC) device

| **Control** | **Olz (50 µM)** | **CGA (50µM)** | **CGA/Olz (50 µM)** | **Met (50 µM)** | **Met/Olz (50 µM)** |
| --- | --- | --- | --- | --- | --- |
| 100 | 127.95 | 57.06 | 79.84 | 87.96 | 86.85 |
| 100 | 138.07 | 62.32 | 51.02 | 79.29 | 113.75 |
| 100 | 124.82 | 74.43 | 71.09 | 77.72 | 84.26 |

2- Quantitative Oil Red O assay of intracellular lipid content of different treatment conditions on HepG2 cells in the perfusion-based co-culture (PBCC) device

| **Control** | **Olz (50 µM)** | **CGA (50µM)** | **CGA/Olz (50 µM)** | **Met (50 µM)** | **Met/Olz (50 µM)** |
| --- | --- | --- | --- | --- | --- |
| 100 | 183.541 | 66.261 | 114.329 | 133.015 | 124.334 |
| 100 | 153.659 | 67.344 | 83.825 | 82.454 | 84.884 |
| 100 | 153.565 | 78.518 | 85.701 | 99.762 | 103.665 |

3- Quantitative Oil Red O assay of intracellular lipid content of different treatment conditions on 3T3-L1 in 96-well plate

| **Control** | **Olz (50 µM)** | **CGA (50µM)** | **CGA/Olz (50 µM)** | **Met (50 µM)** | **Met/Olz (50 µM)** |
| --- | --- | --- | --- | --- | --- |
| 100 | 177.714 | 111.683 | 94.1459 | 107.390 | 122.259 |
| 100 | 197.572 | 134.785 | 109.596 | 102.215 | 118.444 |
| 100 | 121.461 | 119.267 | 88.348 | 76.965 | 138.076 |

4- Quantitative Oil Red O assay of intracellular lipid content of different treatment conditions on HepG2 cells in 96-well plate

| **Control** | **Olz (50 µM)** | **CGA (50µM)** | **CGA/Olz (50 µM)** | **Met (50 µM)** | **Met/Olz (50 µM)** |
| --- | --- | --- | --- | --- | --- |
| 100 | 121.939 | 100.447 | 111.085 | 92.5899 | 111.765 |
| 100 | 119.673 | 113.811 | 124.311 | 88.694 | 115.864 |
| 100 | 114.805 | 115.101 | 107.122 | 80.754 | 123.918 |

B- Glucose concentration

1- Glucose concentration in the culture medium for the perfusion-based co-culture (PBCC) device

| **Control** | **Olz (50 µM)** | **CGA (50µM)** | **CGA/Olz (50 µM)** | **Met (50 µM)** | **Met/Olz (50 µM)** |
| --- | --- | --- | --- | --- | --- |
| 100 | 134.568 | 88.696 | 111.710 | 79.299 | 128.649 |
| 100 | 148.265 | 85.125 | 99.631 | 105.732 | 136.023 |
| 100 | 140.854 | 76.955 | 103.811 | 100 | 120 |

2- Glucose concentration in the culture medium for 3T3-L1 cells in a 96-well plate

| **Control** | **Olz (50 µM)** | **CGA (50µM)** | **CGA/Olz (50 µM)** | **Met (50 µM)** | **Met/Olz (50 µM)** |
| --- | --- | --- | --- | --- | --- |
| 100 | 86.07 | 59.02 | 81.97 | 94.26 | 81.15 |
| 100 | 100 | 78 | 99 | 105 | 85 |
| 100 | 90 | 72.73 | 72.7 | 100 | 73 |

3- Glucose concentration in the culture medium for HepG2 cells in a 96-well plate

| **Control** | **Olz (50 µM)** | **CGA (50µM)** | **CGA/Olz (50 µM)** | **Met (50 µM)** | **Met/Olz (50 µM)** |
| --- | --- | --- | --- | --- | --- |
| 100 | 65.97 | 76.05 | 58.82 | 66.81 | 73.53 |
| 100 | 80 | 92.5 | 75 | 82.5 | 90 |
| 100 | 83.33 | 95.24 | 65.24 | 80.95 | 90.48 |

C- Triglyceride (TG) concentration

1- Triglyceride (TG) concentration in the culture medium for the PBCC device

| **Control** | **Olz (50 µM)** | **CGA (50µM)** | **CGA/Olz (50 µM)** | **Met (50 µM)** | **Met/Olz (50 µM)** |
| --- | --- | --- | --- | --- | --- |
| 100 | 98 | 106.9 | 69.8 | 97.7 | 79 |
| 100 | 120.9 | 98 | 93 | 70 | 98 |
| 100 | 130 | 97.67 | 111.6 | 80 | 92 |

2- Triglyceride (TG) concentration in the culture medium for 3T3-L1 cells in a 96-well plate

| **Control** | **Olz (50 µM)** | **CGA (50µM)** | **CGA/Olz (50 µM)** | **Met (50 µM)** | **Met/Olz (50 µM)** |
| --- | --- | --- | --- | --- | --- |
| 100 | 42.9 | 85.7 | 43 | 71 | 35.7 |
| 100 | 71.4 | 114.3 | 36 | 64 | 57 |
| 100 | 60 | 121 | 40 | 70 | 60 |

3- Triglyceride (TG) concentration in the culture medium for HepG2 cells in a 96-well plate

| **Control** | **Olz (50 µM)** | **CGA (50µM)** | **CGA/Olz (50 µM)** | **Met (50 µM)** | **Met/Olz (50 µM)** |
| --- | --- | --- | --- | --- | --- |
| 100 | 128.6 | 42.8 | 72 | 85.7 | 107 |
| 100 | 133 | 58 | 108 | 100 | 150 |
| 100 | 130.8 | 46.15 | 92.3 | 115.4 | 107.7 |
