# Supplementary material for: A pumpless liver-adipose model for studying metabolic dysfunction and drug responses
Source: PLoS One. 2026 May 8;21(5):e0345524. doi: 10.1371/journal.pone.0345524 (PMC13155575; doi:10.1371/journal.pone.0345524)
Supplement: Attachment — Submitted filename: Liver adipose.docx [file pone.0345524.s002.docx]

**Reviewer 1**

| **Reviewer Recommendation Term:** | Major Revision |
| --- | --- |
| **Rate Review:** | 0 |
| **Custom Review Question(s):** \| **Response**; **Comments to the Author** 1. Is the manuscript technically sound, and do the data support the conclusions? \| Partly; 2. Has the statistical analysis been performed appropriately and rigorously? \| Yes; 3. Have the authors made all data underlying the findings in their manuscript fully available? \| Yes; 4. Is the manuscript presented in an intelligible fashion and written in standard English? \| Yes; 5. Review Comments to the Author \| The authors present a pumpless, open-top, passive-circulation co-culture device (POCC) composed of four chambers to simulate liver–adipose interactions. HepG2 and 3T3-L1 were cultured in separate compartments and exposed to drug treatments, with metabolic responses evaluated through viability, lipid accumulation, glucose consumption, and extracellular triglyceride (TG) levels. The platform is innovative and shows strong potential as a passive-flow liver–adipose screening system. With additional functional validation and clearer justification of experimental design, the work could become a valuable contribution to metabolic disease modeling and early drug response studies. The manuscript would be strengthened by addressing the following points: Major Comments Are there any leakage or delamination issues after device assembly or during perfusion? If so, how were these assessed and mitigated? The authors should specify how frequently media were exchanged or replenished and justify that. Passive-flow metabolic models are sensitive to nutrient depletion and waste accumulation. Section 2.5 states that cells were cultured in T25 flasks with high-glucose DMEM + 10% FBS + 1% Pen/Strep. The authors should confirm whether both cell types (HepG2 and 3T3-L1) were cultured under identical conditions, or justify any differences. Moreover, 3T3-L1 are pre-adipocytes. The authors should clearly state whether differentiation into mature adipocytes was performed before drug exposure. If not, this limits the metabolic relevance of adipokine and lipid-mobilization readouts. The authors should justify why hepatocytes and adipocytes were cultured in separate chambers rather than together and having 4 representative chambers. If the intent is a liver–adipose interaction model, this should be explicitly stated earlier and supported with a proper rationale. The 1:1 ratio of 3T3-L1 : HepG2 cells should be justified. Before positioning the system as a disease model or an alternative to animal testing, the hepatic compartment should be validated for core liver metabolic/synthetic function under perfusion, even at a phenotypic/directional level. At minimum, I recommend assessing: • Albumin secretion (e.g., in device vs static control in 96-well plate) • Urea production trends The authors should expand discussion on why passive-flow/perfused culture better mimics in vivo compared to static systems, ideally correlating the device phenotype with in vivo hepatic vascular and metabolic behavior. In addition to bright-field microscopy, the study would benefit from live/dead fluorescence imaging (e.g., Calcein-AM + PI) to visually support viability under perfusion, as well as clearer microscopic evidence of morphology changes in each chamber in Figure 5. Minor Comments The paragraph describing serial dilution with <1% DMSO in Section 2.6 is repeated and should be removed for clarity. Section 2.7 states that 3×10³ cells/well were seeded, while Figure 3 reports 3×10³ cells/mL. The correct units should be verified and standardized. The authors reference Equation (1) for calculating flow rate, but no equation was found in the manuscript. This should be corrected. Future Work Suggestions Transitioning the co-culture into 3D spheroids would significantly improve physiological relevance. This could be achieved by: o Non-adhesive well surface treatment o Increased cell density o Incorporation of ECM-based hydrogels to support 3D tissue organization.; 6. PLOS authors have the option to publish the peer review history of their article ([what does this mean?](https://journals.plos.org/plosone/s/editorial-and-peer-review-process#loc-peer-review-history)). If published, this will include your full peer review and any attached files. \| **Yes:** Hossein Abolhassani; **Confidential to Editor** 1. Do you have any potential or perceived competing interests that may influence your review? \| I have no competing interests; 2. Did you receive any assistance in preparing this review \| No; 3. If accepted, do you think this submission should be highlighted on the PLOS ONE website? \|; **Do you want to get recognition for this review on** [**a Web of Science researcher profile**](https://clarivate.com/products/scientific-and-academic-research/research-discovery-and-workflow-solutions/researcher-profiles/)**?** \| Yes | |

**Reviewer 2**

| **Reviewer Recommendation Term:** | Minor Revision |
| --- | --- |
| **Rate Review:** | 0 |
| **Custom Review Question(s):** \| **Response**; **Comments to the Author** 1. Is the manuscript technically sound, and do the data support the conclusions? \| Yes; 2. Has the statistical analysis been performed appropriately and rigorously? \| Yes; 3. Have the authors made all data underlying the findings in their manuscript fully available? \| Yes; 4. Is the manuscript presented in an intelligible fashion and written in standard English? \| Yes; 5. Review Comments to the Author \| The work is good. The author should include some genomic studies and in vivo studies in mouse model. If in vivo study in animal model is not possible then it may be discussed with appropriate work previously done; 6. PLOS authors have the option to publish the peer review history of their article ([what does this mean?](https://journals.plos.org/plosone/s/editorial-and-peer-review-process#loc-peer-review-history)). If published, this will include your full peer review and any attached files. If you choose “no”, your identity will remain anonymous but your review may still be made public. **Do you want your identity to be public for this peer review?** For information about this choice, including consent withdrawal, please see our [Privacy Policy](https://www.plos.org/privacy-policy). \| No; **Confidential to Editor** 1. Do you have any potential or perceived competing interests that may influence your review? \| The work is good. The author should include some genomic studies and in vivo studies in mouse model. If in vovo study in animal model is not possible then it may be discussed with appropriate work previously done. After minor revision it may be accepted; 2. Did you receive any assistance in preparing this review (e.g. from a post-doc or graduate student)? If yes, please include their name below. \| no; 3. If accepted, do you think this submission should be highlighted on the PLOS ONE website? PLOS ONE does not evaluate manuscripts based on perceived significance or readership. We aim to provide tools for readers to filter and evaluate our publications. (optional) \| No; **Do you want to get recognition for this review on** [**a Web of Science researcher profile**](https://clarivate.com/products/scientific-and-academic-research/research-discovery-and-workflow-solutions/researcher-profiles/)**?** \| Yes | |
