# Supplementary material for: A pumpless liver-adipose model for studying metabolic dysfunction and drug responses
Source: PLoS One. 2026 May 8;21(5):e0345524. doi: 10.1371/journal.pone.0345524 (PMC13155575; doi:10.1371/journal.pone.0345524)
Supplement: Attachment — Submitted filename: Response to Reviewers- 13 Feb.docx [file pone.0345524.s004.docx]

**Dear Editor-in-Chief,**

**We thank the editor and the reviewers for their careful, constructive, and insightful comments, which significantly improved the quality and clarity of the manuscript. All comments and queries were responded in a point-by-point manner, and the corresponding revisions have been incorporated into the revised manuscript and highlighted using Track Changes.**

**Below, we provide a detailed, point-by-point response to all reviewer comments.**

**Editor:**

**1. Please ensure that your manuscript meets PLOS ONE's style requirements, including those for file naming.**

Response: We have reviewed the manuscript in detail and confirmed that it adheres to the PLOS ONE formatting and style guidelines, as outlined in the provided templates. The manuscript files have been prepared accordingly.

**2. Thank you for stating the following financial disclosure: “The authors are grateful to the Vice Chancellor of Research, Mashhad University of Medical Sciences (No. 971985), Mashhad, Iran for financial support”**

**Please state what role the funders took in the study.  If the funders had no role, please state: "The funders had no role in study design, data collection and analysis, decision to publish, or preparation of the manuscript." If this statement is not correct you must amend it as needed. Please include this amended Role of Funder statement in your cover letter; we will change the online submission form on your behalf.**

Response: We confirm that the funders had no role in the study design, data collection and analysis, decision to publish, or preparation of the manuscript. We have included this statement in the cover letter as requested.

**3. We note that your Data Availability Statement is currently as follows: All relevant data are within the manuscript and in Supporting Information files. Please confirm at this time whether or not your submission contains all raw data required to replicate the results of your study. Authors must share the “minimal data set” for their submission. PLOS defines the minimal data set to consist of the data required to replicate all study findings reported in the article, as well as related metadata and methods (**[**https://journals.plos.org/plosone/s/data-availability#loc-minimal-data-set-definition**](https://journals.plos.org/plosone/s/data-availability#loc-minimal-data-set-definition)**)....**

Response: Thank you for this clarification. The manuscript reports summary data (means and variability measures) based on raw data derived from experiments, in form of figures and table. No additional raw datasets beyond those reported were generated or analyzed. Raw data will be available upon request. If needed, please revise the Data Availability Statement to accurately reflect the data included in this study, as follows: “Data will be available by the authors upon a request.”

**4. If the reviewer comments include a recommendation to cite specific previously published works, please review and evaluate these publications to determine whether they are relevant and should be cited. There is no requirement to cite these works unless the editor has indicated otherwise.**

Response: The reviewers did not recommend citation of any specific previously published works; therefore, no additional references were required.

**5. Additional Editor Comments: Authors need to respond to the all the queries raised by reviewers**

Response: We carefully reviewed all reviewers’ comments. All reviewers’ queries were fully addressed, and corresponding revisions were made in the manuscript where appropriate.

**Reviewer 1**

**1. Are there any leakage or delamination issues after device assembly or during perfusion? If so, how were these assessed and mitigated?**

Response: We thank the reviewer for raising this important point. This procedure has been added to Section 2.6 of the revised manuscript (lines 195 and 201): “Prior to cell seeding, multiple microfluidic devices were fabricated and pre-tested using deionized water for at least 24 hours to assess structural integrity and potential leakage. Only devices showing no signs of leakage were selected for cell seeding and subsequent experiments. Throughout the cell culture period, devices were routinely monitored for leakage or delamination. If any structural failure was observed, the affected device was excluded from the study and replaced with a pre-tested backup device prepared for the corresponding experimental group. Only data obtained from intact, leak-free devices were included in the final analysis.”

**2. The authors should specify how frequently media were exchanged or replenished and justify that. Passive-flow metabolic models are sensitive to nutrient depletion and waste accumulation.**

Response: To preserve accumulated metabolic signals and prevent dilution of secreted factors, the culture medium was not routinely exchanged during the experiments. Preliminary optimization studies were performed to identify a cell seeding density that could maintain viability and metabolic activity over the four-day culture period without frequent medium replacement. Various seeding densities for HepG2 and 3T3-L1 cells were tested, and a density of 3,000 cells per compartment was selected as it ensured stable metabolic activity while avoiding nutrient depletion and excessive waste accumulation. To clarify this point, we have added the following description to Section 2.7 of the revised manuscript (lines 219 and 223): “No complete medium exchange was performed during the culture period. In a limited number of cases, approximately 10% (120 µl) of the total medium volume was carefully removed and replaced with fresh medium after 48 hours. This partial replenishment strategy was employed to support cell viability while minimizing dilution of accumulated metabolic signals.”

**3. Section 2.5 states that cells were cultured in T25 flasks with high-glucose DMEM + 10% FBS + 1% Pen/Strep. The authors should confirm whether both cell types (HepG2 and 3T3-L1) were cultured under identical conditions, or justify any differences.**

Response: Both HepG2 and 3T3-L1 cells were routinely cultured under identical conditions prior to seeding into the microfluidic devices, using high-glucose DMEM supplemented with 10% fetal bovine serum (FBS) and 1% penicillin–streptomycin. No differences in basal culture conditions were applied between the two cell types. This clarification has now been added to Section 2.5 of the revised manuscript (lines 168 and 172): “Both HepG2 and 3T3-L1 cells …. No differences in basal culture conditions were applied between the two cell types.”

**4. 3T3-L1 are pre-adipocytes. The authors should clearly state whether differentiation into mature adipocytes was performed before drug exposure. If not, this limits the metabolic relevance of adipokine and lipid-mobilization readouts.**

Response: We thank the reviewer for this important comment. In the present study, 3T3-L1 cells were used in the pre-adipocyte stage and were not differentiated into mature adipocytes prior to drug exposure. This was a deliberate design choice aimed at investigating early lipid accumulation and adipogenic responses of adipose precursor cells under the influence of hepatocyte-derived paracrine signaling within the co-culture system. Lipid accumulation was assessed using Oil Red O staining, a well-established marker of adipogenic and lipogenic activity in 3T3-L1 pre-adipocytes.

Increased triglyceride accumulation observed in 3T3-L1 pre-adipocytes exposed to hepatocyte-conditioned environments is consistent with previous reports demonstrating enhanced lipid accumulation in 3T3-L1 cells treated with hepatocyte-conditioned media under metabolic stress conditions (1). These findings support the relevance of the selected model for studying early liver–adipose metabolic crosstalk.

We acknowledge that the use of pre-adipocytes limits conclusions regarding mature adipocyte-specific functions. This limitation, together with relevant supporting literature, has now been explicitly discussed in the revised discussion section (lines 527-535): “Another limitation of the present study is that 3T3-L1 cells were used in the pre-adipocyte stage rather than fully differentiated adipocytes. While this approach enabled the investigation of early lipid accumulation and liver–adipose crosstalk within the microfluidic platform, it limits conclusions regarding mature adipocyte-specific functions such as adipokine secretion.

Notably, previous studies have shown that hepatocyte-derived conditioned media and extracellular vesicles can influence adipogenic and lipogenic responses in 3T3-L1 pre-adipocytes even in the absence of standard differentiation protocols [80]. Although EV-mediated signaling was not directly assessed in the present study, such paracrine mechanisms may contribute to the enhanced lipid accumulation observed in the co-culture system and warrant further investigation.” In addition, we have clarified in Section 2.5 of the Methods (lines 172-174): “3T3-L1 cells were used in the pre-adipocyte state and they were not differentiated into mature adipocytes prior to drug exposure.”

**5. The authors should justify why hepatocytes and adipocytes were cultured in separate chambers rather than together and having 4 representative chambers. If the intent is a liver–adipose interaction model, this should be explicitly stated earlier and supported with a proper rationale.**

Response: In the initial version of our model, hepatic and adipose cells were cultured in a simplified, compartmentalized configuration to establish a controllable and reproducible platform for studying inter-tissue metabolic communication. Accordingly, we have expanded the discussion section (lines 410-427) to further describe the design rationale and advantages of the POCC platform, particularly in terms of enabling dynamic inter-tissue communication, low-shear passive perfusion, and direct comparison with conventional static co-culture systems.

“Cell–cell interactions, particularly between metabolically distinct tissues, have gained increasing attention due to their physiological relevance in both fundamental and translational biomedical research [51]. The POCC platform developed in this study consists of four interconnected culture chambers, enabling simultaneous co-culture of different cell types and facilitating biochemical communication between tissue compartments.

Passive medium circulation was achieved using a rotator-based system, which allowed controlled and homogeneous exchange of soluble factors between chambers while maintaining low shear stress conditions compatible with cell viability. The geometric design of the channels and chambers ensured uniform flow without obstruction, minimized bubble formation, and supported efficient gas exchange through the open-top configuration. Importantly, the chamber dimensions were designed to be comparable to those of a standard 96-well plate, enabling direct comparison between monoculture and co-culture conditions. While the present study focused on liver–adipose interactions, the modular design of the platform allows scalability to additional tissue compartments, providing flexibility for modeling more complex inter-organ communication in future studies. Unlike conventional static co-culture systems such as Transwell inserts, which rely on diffusion-limited exchange and lack dynamic medium circulation, the POCC platform enables continuous biochemical communication under controlled flow conditions, thereby improving the physiological relevance of inter-tissue metabolic interactions.”

**6. The 1:1 ratio of 3T3-L1: HepG2 cells should be justified.**

Response: We thank the reviewer for this important comment. Defining a physiologically exact hepatic-to-adipose cell ratio that can be directly translated into an in vitro co-culture system is challenging, as adipose tissue mass, distribution, and metabolic activity are highly dynamic and vary with nutritional status, body composition, and disease conditions. Consequently, in liver–adipose co-culture models, the primary objective is not to replicate a fixed in vivo tissue proportion, but rather to enable controlled investigation of paracrine metabolic communication between compartments.

In the present study, a 1:1 seeding ratio of 3T3-L1 and HepG2 cells was selected based on preliminary optimization experiments and to provide balanced bidirectional signaling without dominance of either cell population. This ratio was chosen as a practical and reproducible starting point for this proof-of-concept platform and has now been discussed in the revised Discussion section (lines 437-449).

“Although physiological relevance is an important consideration in multi-tissue in vitro models, translating in vivo hepatic–adipose tissue proportions into a fixed in vitro cell ratio remains challenging [54]. Adipose tissue mass and metabolic activity are highly dynamic and vary substantially with nutritional status, body composition, and disease state, and different adipose depots contribute differently to systemic metabolism. These factors complicate the definition of a in vivo tissue ratio and highlight that simple mass-based ratios may not fully reflect physiological dynamics [55,56]. As a result, liver–adipose co-culture systems generally prioritize capturing paracrine metabolic communication rather than replicating exact in vivo tissue proportions. In this study, a 1:1 ratio of 3T3-L1 to HepG2 cells was therefore selected as a balanced and reproducible starting point to support bidirectional metabolic signaling between compartments, while maintaining experimental simplicity and robustness in this proof-of-concept platform. In Future studies, other ratios of hepatic to adipose cells could be investigated according to the scope of a study.”

**7. Before positioning the system as a disease model or an alternative to animal testing, the hepatic compartment should be validated for core liver metabolic/synthetic function under perfusion, even at a phenotypic/directional level. At minimum, I recommend assessing:**

**• Albumin secretion (e.g., in device vs static control in 96-well plate)**

**• Urea production trends**

Response: We thank the reviewer for this valuable suggestion. We agree that direct assessment of hepatic synthetic function, including albumin secretion and urea production, is essential for full validation of the hepatic compartment, particularly when positioning the system as a disease-relevant or animal-alternative model.

The present study was designed as a proof-of-concept platform to investigate liver–adipose metabolic interactions and drug-induced metabolic alterations under passive perfusion, rather than to establish a fully validated liver disease model. Accordingly, direct measurements of albumin and urea production were not included and are now explicitly acknowledged as a limitation of the current work.

This limitation and the incorporation of established hepatic functional markers, such as albumin and urea, as part of future validation studies have been clearly stated in the revised Discussion section (lines 515-526): “Albumin secretion and urea synthesis are widely accepted liver-specific functional markers for validating hepatic synthetic activity in vitro liver models and under dynamic and perfused culture conditions in liver-on-chip systems [78]. Such measurements are commonly incorporated in later-stage validation studies once platform feasibility, stability, and metabolic responsiveness have been established.

In the present study, the primary objective was not to establish a fully validated liver disease model, but rather to develop and apply a pumpless co-culture platform to investigate inter-tissue metabolic communication and drug-induced metabolic alterations between liver and adipose compartments. Accordingly, direct functional validation assays of hepatic synthetic activity, such as albumin and urea production, were not included and are acknowledged as a limitation of the current work. Future studies will incorporate these established functional endpoints to further validate hepatic performance within the POCC system, as demonstrated in prior perfused HepG2-based microfluidic models [79].”

**8. The authors should expand discussion on why passive-flow/perfused culture better mimics in vivo compared to static systems, ideally correlating the device phenotype with in vivo hepatic vascular and metabolic behavior.**

Response: We thank the reviewer for this insightful comment. We have expanded the Discussion (lines 493-514) to further clarify why the passive-flow/perfused culture better mimics in vivo conditions compared to static systems.

“One of the notable aspects of this study was the direct comparison between cellular responses obtained from conventional static 96-well plate cultures and the POCC microfluidic platform. In static 96-well cultures, Olz-, CGA-, and Met-induced changes in lipid, glucose, and triglyceride accumulation were heterogeneous across individual cell types and, in some cases, even contradictory, showing limited concordance with patterns reported in in vivo models [50]. In contrast, in the microfluidic chip system, the observed alterations in intracellular glucose and lipid levels were more consistent with findings from animal studies. These findings suggest that, in the current POCC system, intercellular communication facilitated by interconnected channels and intermittent passive perfusion under low shear stress may enhance physiological relevance by improving metabolite distribution and exchange between culture compartments. This configuration allows soluble factors secreted by hepatic and adipose cells to circulate between compartments without substantial washout or dilution.

Although this system does not replicate vascular perfusion or hepatic sinusoidal architecture, the low-shear, periodic circulation of culture medium may partially support more uniform exposure of hepatocytes to soluble metabolic signals, thereby approximating certain aspects of the hepatic metabolic microenvironment observed in vivo. Moreover, the compartmentalized configuration enables hepatic and adipose cells to maintain tissue-specific phenotypes while supporting paracrine and endocrine-like communication through shared medium circulation. Such functional coupling more closely approximates liver–adipose metabolic crosstalk observed in vivo, even in the absence of continuous perfusion or endothelial-lined vasculature. Consequently, drug-induced metabolic responses in the POCC system—particularly changes in intracellular lipid accumulation and glucose handling—were more physiologically aligned with in vivo observations than those obtained under static culture conditions.

**9. In addition to bright-field microscopy, the study would benefit from live/dead fluorescence imaging (e.g., Calcein-AM + PI) to visually support viability under perfusion, as well as clearer microscopic evidence of morphology changes in each chamber in Figure 5.**

Response: We thank the reviewer for this valuable suggestion. We agree that live/dead fluorescence staining (e.g., Calcein-AM/PI) would provide additional qualitative visual confirmation of cell viability under perfusion conditions.

In the present study, cell viability was primarily assessed using quantitative metabolic assays (Alamar Blue), which allowed comparative evaluation of viability across experimental groups and culture platforms. We have also exhibited the description of morphological observations in Figure 5 to enhance clarity.

Representative bright-field images showing preserved cell morphology and attachment in each chamber after 4 days of culture. Due to experimental scope and resource limitations, live/dead fluorescence imaging was not performed.

**10. The paragraph describing serial dilution with <1% DMSO in Section 2.6 is repeated and should be removed for clarity. Section 2.7 states that 3×10³ cells/well were seeded, while Figure 3 reports 3×10³ cells/mL. The correct units should be verified and standardized. The authors reference Equation (1) for calculating flow rate, but no equation was found in the manuscript. This should be corrected.**

Response: The repeated paragraph describing serial dilution with <1% DMSO in Section 2.6 has been removed in the revised manuscript to improve clarity.

Regarding cell seeding density, we have verified the correct units and standardized them throughout the manuscript. The correct seeding density is 3 × 10³ cells per well, and this has now been consistently reflected in both the text and Figure 3. Finally, we acknowledge that Equation (1) was unintentionally referenced without being included in the manuscript. This error was corrected by removing the reference to Equation (1) in the revised Methods section.

**Reviewer 2**

**1. The work is good. The author should include some genomic studies and in vivo studies in mouse model. If in vivo study in animal model is not possible then it may be discussed with appropriate work previously done.**

Response: We thank the reviewer for the positive evaluation of our work and for this valuable suggestion. With respect to in vivo validation, we note that the metabolic effects investigated in the current study have been previously examined by our group in a rat model, and the corresponding results have been published “Exploring the therapeutic potential of chlorogenic acid in alleviating olanzapine-induced metabolic syndrome in rats: a key role of hypothalamic satiety proteins”. The key findings from that animal study have been summarized and appropriately cited in the Discussion (lines: 406-407): “Our pervious animal study showed that Olz increased serum glucose and TG levels and visceral fat, and CCA and Met reduced these metabolic effects in that study [50].” While genomic and in vivo validation would provide additional mechanistic insight, these approaches were beyond the scope of the current study. We have expanded the discussion using relevant published genomic and animal studies including our previous animal study as reflected in the main draft as follows:

(lines: 462-469): “…. In animal studies, CGA has been found to reduce free fatty acids, TG, and plasma cholesterol. It also increased the HDL-cholesterol/total cholesterol ratio compared to that in the high-fat control group [72]. Pervious our study demonstrated that CGA supplementation could effectively reduce Olz-induced weight gain and metabolic abnormalities by affecting the hypothalamic path ways involved in appetite. CGA reversed the upregulation of NPY and pAMPK protein expression, as well as the downregulation of POMC protein expression caused by Olz treatment, CGA demonstrated comparable efficacy to Met in treating Olz-induced metabolic syndrome, making it a promising supplement for this purpose [50].”

(lines: 482-492): “…. Given the observed changes in glucose, TG, and lipid accumulation levels in this study, would suggest that Olz, CGA, and Met may have a great effect on critical metabolic pathways. Previous studies have reported that Olz induces adipogenesis through the SREBP-1 pathway in 3T3-L1 cells, leading to increased lipogenesis and lipid deposition in these cells [65]. It also gets involve impairment in insulin signaling to have increment in glucose and lipid levels [60]. A recent study demonstrated that CGA and its isomers effectively mitigated NAFLD in HepG2 cells. This effect was achieved through a reduction in lipid accumulation and TG levels [76]. CGA has been reported to exert anti-lipogenic effects in 3T3-L1 adipocytes through AMP-activated protein kinase (AMPK) activation. What is more prevention of fatty acid synthesis via suppression of acetyl-CoA carboxylase and peroxisome proliferator-activated receptor gamma (PPARγ) [76,77]. The mechanism of metformin also involves AMPK activation, which enhances glucose uptake and inhibits lipogenesis [8].”

1.	Zhao Y, Zhao M, Jiang S, Wu J, Liu J, Zhang J, et al. Liver governs adipose remodelling via extracellular vesicles in response to lipid overload. Nat Commun [Internet]. 2020; Available from: http://dx.doi.org/10.1038/s41467-020-14450-6
